# A convenient scheme for coupling a finite element curvilinear mesh to a finite element voxel mesh: application to the heart

**DOI:** 10.1186/1475-925X-5-60

**Published:** 2006-11-17

**Authors:** Bruce Hopenfeld

**Affiliations:** 1 Angel Medical Systems, Tinton Falls, New Jersey, USA

## Abstract

**Background:**

In some cases, it may be necessary to combine distinct finite element meshes into a single system. The present work describes a scheme for coupling a finite element mesh, which may have curvilinear elements, to a voxel based finite element mesh.

**Methods:**

The method is described with reference to a sample problem that involves combining a heart, which is defined by a curvilinear mesh, with a voxel based torso mesh. The method involves the creation of a temporary (scaffolding) mesh that couples the outer surface of the heart mesh to a voxel based torso mesh. The inner surface of the scaffolding mesh is the outer heart surface, and the outer surface of the scaffolding mesh is defined by the nodes in the torso mesh that are nearest (but outside of) the heart. The finite element stiffness matrix for the scaffolding mesh is then computed. This stiffness matrix includes extraneous nodes that are then removed, leaving a coupling matrix that couples the original outer heart surface nodes to adjacent nodes in the torso voxel mesh. Finally, a complete system matrix is assembled from the pre-existing heart stiffness matrix, the heart/torso coupling matrix, and the torso stiffness matrix.

**Results:**

Realistic body surface electrocardiograms were generated. In a test involving a dipole embedded in a spherical shell, relative error of the scheme rapidly converged to slightly over 4%, although convergence thereafter was relatively slow.

**Conclusion:**

The described method produces reasonably accurate results and may be best suited for problems where computational speed and convenience have a higher priority than very high levels of accuracy.

## Background

In some cases, it may be necessary to combine distinct finite element meshes into a single system. For example, the task that motivated this work involved integrating a curvilinear mesh[1] of the Auckland canine heart[2] with a voxel mesh of a human shaped torso. The heart mesh described in [1] was created to perform simulations on a stand alone heart. Fischer et al. [3] describe an elegant technique for coupling a stand alone heart model to a torso surface via the boundary element method, but this scheme is not applicable where it is desired to know the intra-thoracic potential distribution, which requires a full volume mesh.

Many types of volume meshing strategies exist[4]. Meshes can generally be divided into two categories, structured meshes and unstructured meshes. Structured meshes, which include voxel meshes, are characterized by interior nodes that are connected to the same number of nodes. In contrast, the internal nodes in an unstructured mesh may be connected to different numbers of nodes. Structured meshes are generally easy to work with because of their regularity. However, it may be difficult to conform a structured mesh to an irregular boundary, a task which is easier with an unstructured mesh.

One approach for coupling a voxel mesh to a mesh with an irregular boundary (e.g. the outer heart surface) involves individual treatment of the voxel elements that intersect the boundary. This scheme, known as the Cartesian cut cell method[5], has the drawback of being relatively complex and therefore likely to be difficult to implement. Another coupling technique involves extruding prism elements from a triangular surface mesh (e.g. the outer heart surface) in the surface normal direction, and then coupling the prism elements to Cartesian elements[6]. Again, the disadvantage of this approach is that it is relatively complicated.

The present work describes a technique that is a variant of the hybrid Cartesian/prism scheme, with a novel method for coupling the prism elements to the voxel elements. The scheme is easy to code, computes quickly, requires little or no user supervision, and has all the advantages associated with voxel meshes. The main drawbacks of the scheme are relatively uneven and slow convergence after a certain degree of accuracy has been reached. One additional limitation is that the scheme is most suitable for geometries which are reasonably well characterized by a spherical harmonic expansion.

## Methods

### Outline

**Figure 1:**
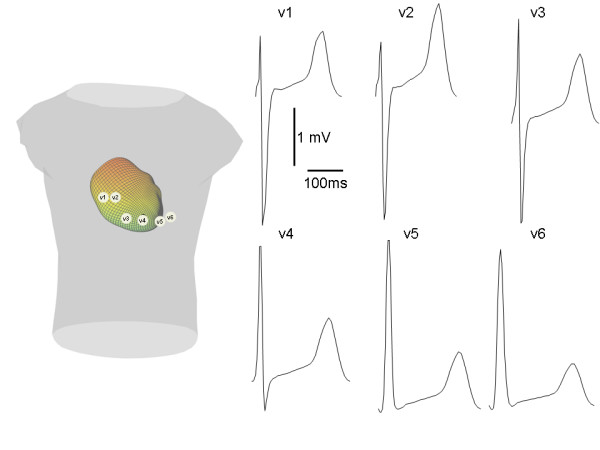
Sample problem. The heart/torso system is shown in the left panel. The right panel shows corresponding simulated electrograms at the sites designated in the left panel.

The present scheme will be described with reference to a particular problem that involves coupling a previously meshed heart[1] to a torso, as shown in the left hand panel in Figure 1. The goal is to solve for the extracellular potential (*V*_e_) throughout the heart and the potential outside of the heart (*V*_o_) in the elliptical equation[7]

∇•(*D*_*i *_+ *D*_*e*_)∇*V*_*e *_= -∇•(*D*_*i*_)∇*V*_*t*_; within heart muscle

∇•(*σ*_*o*_)*V*_*o *_= 0; in the torso and blood pools     (1)

where *D*_i _and *D*_e _are the intracellular and extracellular conductivity tensors within heart muscle, σ_o_ represents the conductivity in the torso and blood pools respectively, and *V*_t _are the transmembrane potentials throughout heart muscle. At the interface between the heart and torso, the potential is continuous (V_e _= V_o_), the normal component of the extracellular current is continuous with the normal component of the current through the torso/blood pools (**n**^T ^**D**_e_V_e _= **n**^T ^σ_o _V_o_, where n is the unit normal vector), and there is no flow of intracellular current into the torso/blood pools (**n**^T ^**D**_i_V_i _= 0).

**Figure 2:**
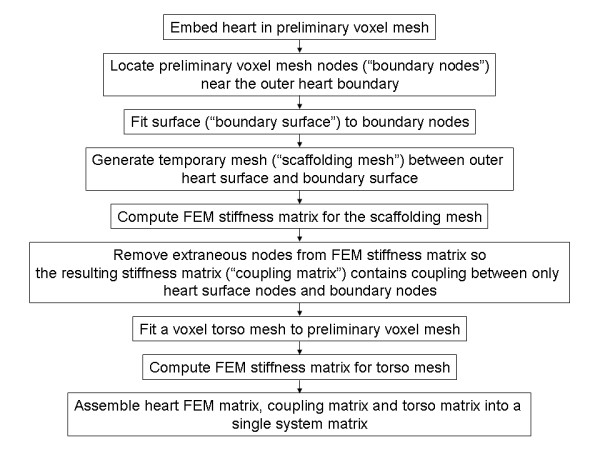
Flow chart of the coupling scheme.

**Figure 3:**
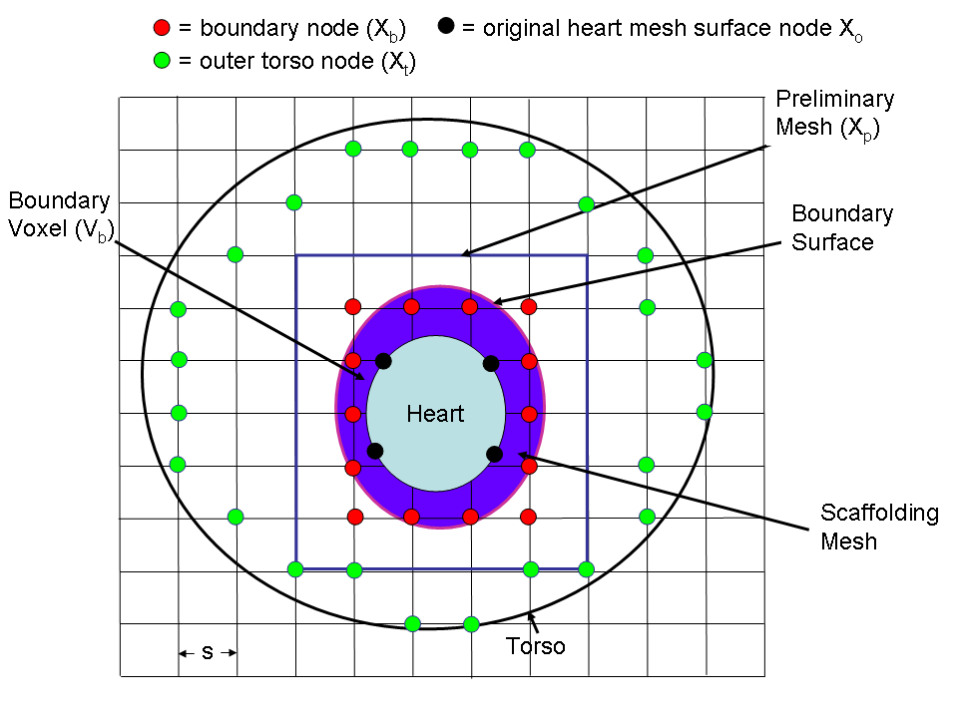
General mesh structure in 2 dimensions. The heart is embedded in a preliminary "scaffolding" voxel mesh, which comprises all of the voxels within the blue rectangle. The preliminary mesh is in turn embedded and aligned with a voxel torso mesh, whose outer nodes are shown in green. Within the preliminary mesh, the voxels that have nodes both inside and outside of the heart are located, and the nodes in these voxels that are outside of the heart are defined as boundary nodes (X_b_). A surface (the "boundary surface") is fitted to these nodes. A coupling mesh (in purple) between the outer heart surface and the boundary surface is then generated.

Figure 2 is a flow chart of the coupling scheme. The first step of the method involves embedding the heart in a preliminary voxel mesh that is slightly larger than the heart, as indicated in Figure 3. The next step involves locating preliminary voxel mesh nodes ("boundary nodes") that are outside of the heart but close to the heart, as indicated by the red circles in Figure 3. A surface ("boundary surface") is fitted to these boundary nodes. The boundary surface is essentially a projection of the outer heart surface, as shown in Figure 3. A temporary mesh ("scaffolding mesh") is generated between the outer heart surface and the boundary surface and the stiffness matrix for this mesh is computed. The scaffolding mesh is indicated by the purple area in Figure 3. The scaffolding mesh includes extraneous temporary nodes that are then removed from the stiffness matrix, leaving a coupling matrix that couples the pre-existing outer heart surface nodes to the boundary surface voxel nodes. A voxel mesh is generated for the entire torso by adding voxels to the preliminary mesh. The resulting stiffness matrix for the torso nodes is then computed. Finally, a complete system matrix is assembled from the pre-existing heart stiffness matrix, the heart/torso coupling matrix and the torso stiffness matrix.

### Preliminary mesh; boundary extraction

Given the heart surface, a preliminary voxel mesh is generated that is slightly larger than the heart. In the two dimensional example illustrated in Figure 3, the preliminary voxel mesh encloses all of the squares within the blue rectangle. The nodes of this mesh are designated as X_p _= (x_p_, y_p_, z_p_). It will be assumed that the voxels are isotropic (side length = s) and chosen to be defined with respect to some convenient origin. The preliminary voxel mesh should extend for a distance of at least 1s in the x, y and z directions beyond any point on the outer heart surface. The voxel size s is preferably the desired voxel size for the entire torso mesh.

**Figure 4:**
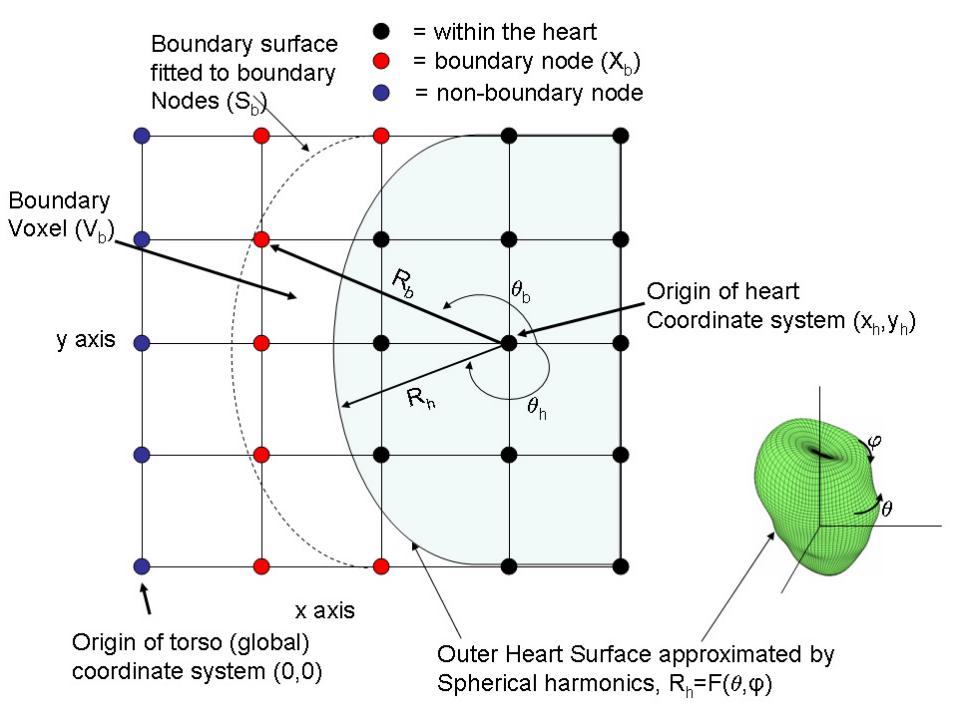
Finding boundary nodes and creating the boundary surface. A convenient origin within the heart serves as the basis for fitting a spherical harmonic expansion to the outer heart surface, which generates a surface characterized by the radius R_h_(θ,Φ). Voxels that have nodes both within and without of this surface are boundary voxels. Within these voxels, the nodes outside of the heart are boundary nodes (red). A surface ("boundary surface") is fitted to these nodes by performing another spherical harmonic expansion about the heart origin. This surface is characterized by the radius R_b_(θ,Φ).

The next step involves locating boundary nodes X_b _⊆ X_p _that are outside of the heart but closest to it, as illustrated in Figures 3 and 4. The boundary nodes are located by finding boundary voxels V_b_, which are defined as those voxels that have at least one node within the heart and at least one node outside of the heart. The boundary nodes X_b _⊆ X_p _are defined as all of the nodes within these voxels V_b _that are outside of the heart.

There are a number of methods for locating the boundary voxels. One convenient method, which was used in the present example, is applicable to any surface (e.g. the heart surface) that may be characterized by spherical harmonics[1]. In this case, the spherical harmonics provide a continuous representation of a surface, and therefore may be used to determine whether any given point is inside or outside of this surface.

More particularly, the outer heart surface S_h _is approximated by R_h _= F(θ,Φ), where R_h _is the radius of the approximate outer heart surface, F is a spherical harmonic polynomial as will be further described below, and θ and Φ are azimuthal and polar angles, respectively. Figure 4 shows a 2-D representation of R_h_. R_h_, θ, and Φ are defined with respect to a convenient origin which is at the point (x_h_, y_h_, z_h_). This origin is preferably chosen such that it results in a good spherical harmonic expansion. The grid points X_p _are translated so their origin is (x_h_, y_h_, z_h_), which results in a set of translated node points X'_p _= (x_p_- x_h_, y_p _- y_h_, z_p _- z_h_). The azimuthal and polar angles θ_p _and Φ_p _(with respect to the (x_h_, y_h_, z_h_) origin) are computed for X'_p_, and then the value F(θ_p_, Φ_p_) is computed, which is the radius of the outer heart surface at the angles θ_p _and Φ_p_. If this radius is greater than the radius (xp−xh)2+(yp−yh)2+(zp−zh)2
 MathType@MTEF@5@5@+=feaafiart1ev1aaatCvAUfKttLearuWrP9MDH5MBPbIqV92AaeXatLxBI9gBaebbnrfifHhDYfgasaacH8akY=wiFfYdH8Gipec8Eeeu0xXdbba9frFj0=OqFfea0dXdd9vqai=hGuQ8kuc9pgc9s8qqaq=dirpe0xb9q8qiLsFr0=vr0=vr0dc8meaabaqaciaacaGaaeqabaqabeGadaaakeaadaGcaaqaaiabcIcaOiabdIha4naaBaaaleaacqWGWbaCaeqaaOGaeyOeI0IaemiEaG3aaSbaaSqaaiabdIgaObqabaGccqGGPaqkdaahaaWcbeqaaiabikdaYaaakiabgUcaRiabcIcaOiabdMha5naaBaaaleaacqWGWbaCaeqaaOGaeyOeI0IaemyEaK3aaSbaaSqaaiabdIgaObqabaGccqGGPaqkdaahaaWcbeqaaiabikdaYaaakiabgUcaRiabcIcaOiabdQha6naaBaaaleaacqWGWbaCaeqaaOGaeyOeI0IaemOEaO3aaSbaaSqaaiabdIgaObqabaGccqGGPaqkdaahaaWcbeqaaiabikdaYaaaaeqaaaaa@4C3A@ of a translated node point, then that point is within the heart. The set of node points within the heart will be denoted by the set X_h _⊆ X_p_.

The above procedure locates the nodes X_h _⊆ X_p _within the heart. The boundary voxels V_b _are then defined as those voxels that have at least one node within the heart (X_h_) and at least one node outside of the heart. The boundary points X_b _are those nodes that are within the boundary voxels but outside of the heart.

### Surface formation

A surface (the boundary surface S_b_) is generated by fitting a spherical harmonic expansion to the points X_b _about the origin (x_h_, y_h_, z_h_). The radii (R_b_) and azimuthal and polar angles θ_b _and Φ_b _are computed for the boundary points, as indicated in Figure 4, which shows only the azimuthal angle. The spherical harmonic expansion of the radii R_b _may be expressed as

Rb=∑i=0N{aiPi(cos⁡ϕb)+∑m=1i(aimcos⁡(mθb)+bimsin⁡(mθb))Pim(cos⁡ϕb)}Rb=P∗C     (2)
 MathType@MTEF@5@5@+=feaafiart1ev1aaatCvAUfKttLearuWrP9MDH5MBPbIqV92AaeXatLxBI9gBaebbnrfifHhDYfgasaacH8akY=wiFfYdH8Gipec8Eeeu0xXdbba9frFj0=OqFfea0dXdd9vqai=hGuQ8kuc9pgc9s8qqaq=dirpe0xb9q8qiLsFr0=vr0=vr0dc8meaabaqaciaacaGaaeqabaqabeGadaaakeaafaqaaeGabaaabaGaemOuai1aaSbaaSqaaiabdkgaIbqabaGccqGH9aqpdaaeWbqaamaacmqabaGaemyyae2aaSbaaSqaaiabdMgaPbqabaGccqWGqbaudaWgaaWcbaGaemyAaKgabeaakiabcIcaOiGbcogaJjabc+gaVjabcohaZHGaciab=v9aQnaaBaaaleaacqWGIbGyaeqaaOGaeiykaKIaey4kaSYaaabCaeaacqGGOaakcqWGHbqydaWgaaWcbaGaemyAaKMaemyBa0gabeaakiGbcogaJjabc+gaVjabcohaZjabcIcaOiabd2gaTjab=H7aXnaaBaaaleaacqWGIbGyaeqaaOGaeiykaKIaey4kaSIaemOyai2aaSbaaSqaaiabdMgaPjabd2gaTbqabaGccyGGZbWCcqGGPbqAcqGGUbGBcqGGOaakcqWGTbqBcqWF4oqCdaWgaaWcbaGaemOyaigabeaakiabcMcaPiabcMcaPiabdcfaqnaaDaaaleaacqWGPbqAaeaacqWGTbqBaaGccqGGOaakcyGGJbWycqGGVbWBcqGGZbWCcqWFvpGAdaWgaaWcbaGaemOyaigabeaakiabcMcaPaWcbaGaemyBa0Maeyypa0JaeGymaedabaGaemyAaKganiabggHiLdaakiaawUhacaGL9baaaSqaaiabdMgaPjabg2da9iabicdaWaqaaiabd6eaobqdcqGHris5aaGcbaacbeGae4Nuai1aaSbaaSqaaiab+jgaIbqabaGccqGH9aqpcqGFqbaucqGHxiIkcqGFdbWqaaGaaCzcaiaaxMaadaqadaqaaiabikdaYaGaayjkaiaawMcaaaaa@87CC@

where N is the number of terms in the spherical harmonic expansion, P_i _is the Legendre function of degree i of the first kind, P_i_^m ^is the associated Legendre function of degree i and order m, a_i_, a_im_, b_im _are coefficients (to be determined), **P **and **C **are matrix and vector representations of the Legendre function values and coefficients, respectively, and **R**_**b **_is a vector representation of the radii R_b_. A least squares fit (**C **= **P**\**R**_**b **_in Matlab notation) results in a set of coefficients C_bs _that define the boundary surface S_b _characterized by R_bs _= F(θ,Φ), where F is the spherical harmonic expansion of order N with coefficients C_bs_, and R_bs _is the radius of a point on the surface S_b _at a given pair of azimuthal and polar angles (θ, Φ).

The appropriate value of N will depend on the geometry of the problem and may be determined empirically. For the heart shown in Figure 1, N = 8 produced good results.

### Scaffolding mesh generation

**Figure 5:**
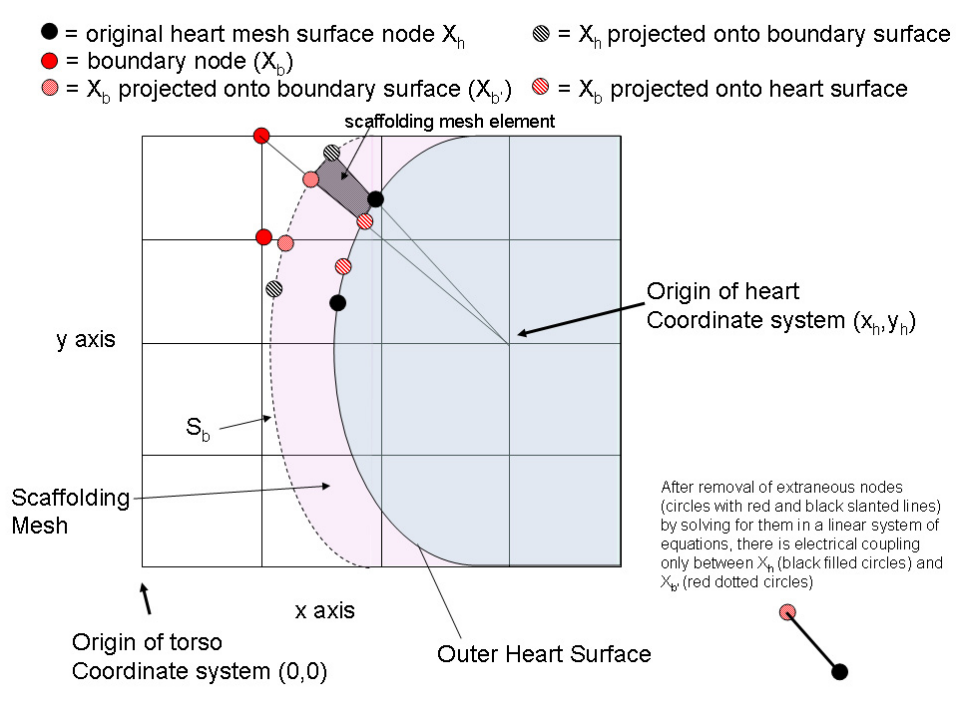
Creating the scaffolding mesh. The boundary nodes (X_b_) are projected onto both the heart surface and the boundary surface, as indicated by the red diagonal line circles and red dotted circles, respectively. The original heart surface nodes are projected on to the boundary surface (black diagonal lined circle). The projected nodes along with the original heart surface node define an element of the scaffolding mesh (shown in black dotted fill for just one element).

The next step involves forming a scaffolding mesh that couples the original heart nodes X_h _to the boundary nodes X_b_. The scaffolding mesh, which is indicated by the purple region in Figure 3, joins the heart surface S_h _to the boundary surface S_b_. With reference to Figure 5, which again shows a two dimensional example of the present scheme, the scaffolding mesh is shown in light purple and one of the scaffolding mesh elements is indicated by a dotted black quadrilateral. The scaffolding mesh provides an approximation of the electrical coupling between the original heart mesh nodes (X_h_, shown as black filled circles) and the boundary nodes (X_b_, shown as red filled circles). The scaffolding mesh is only an estimate of this electrical coupling because the mesh does not include the actual X_b _nodes, but approximations (dotted red circles) of the locations of the boundary nodes (X_b_).

In three dimensions, the scaffolding mesh comprises prismatic elements. Each prismatic element consists of two similar triangles, one on the heart surface S_h _and one on the boundary surface S_b_, whose corresponding vertices are joined to one another_. _The triangles_, _in turn, are formed from combinations of the original heart nodes X_h _and boundary nodes X_b_, and projections of these nodes_._

More particularly, the heart nodes X_h _are projected onto the surface S_b _by a line that passes through the heart nodes and the heart origin (x_h_, y_h_, z_h_), as illustrated in Figure 4. Similarly, the boundary nodes X_b _are projected onto both the surface S_b _and the surface S_h _by a line that passes through these nodes and the heart origin (x_h_, y_h_, z_h_). The projection of the boundary nodes on to S_b _will be referred to as X_b'_. From an implementation standpoint, the projections may be accomplished by calculating the (θ,Φ) pair for any point (about the (x_h_, y_h_, z_h_) origin), and then computing the radius R = F(θ,Φ) for either the heart surface S_h _or boundary surface S_b _from its corresponding spherical harmonic expansion F(θ,Φ).

The result of the mutual projections is two sets of points, one on S_h _and the other on S_b_, such that each point at a given (θ,Φ) on a surface has a corresponding point on the other surface at the same (θ,Φ). Thus, a triangular tessellation of one surface may be used to generate an identical triangular tessellation of the other surface.

There are many ways to tessellate a surface. The approach that was adopted took advantage of the regularity of the (θ,Φ) coordinates of the heart nodes X_h_. These nodes formed a regular grid in the (θ,Φ) coordinates by choice; it is easy to form a such a regular grid when the surface at issue (S_b_) is characterized by spherical harmonics. Because of the cyclicity of the azimuthal angle θ, the original (θ,Φ) grid (-π<=θ<=π) points were augmented such that those points (θ_a_, Φ_a_) with θ_a_<=-4π/5 were identified, and new points with (θ_a_+2*π, Φ_a_) added to the grid. This "wrapped around" grid was triangulated, and the triangles characterized by all three points having θ>2*π were removed. This type of tessellation is generally acceptable only in cases when there are points at both poles (Φ = +/-pi) and there is a reasonably smooth gradation in the Φ values of the points. This condition was satisfied in the present case because the original heart points X_h _were generated via a spherical harmonic expansion with an even division in the Φ direction.

**Figure 6:**
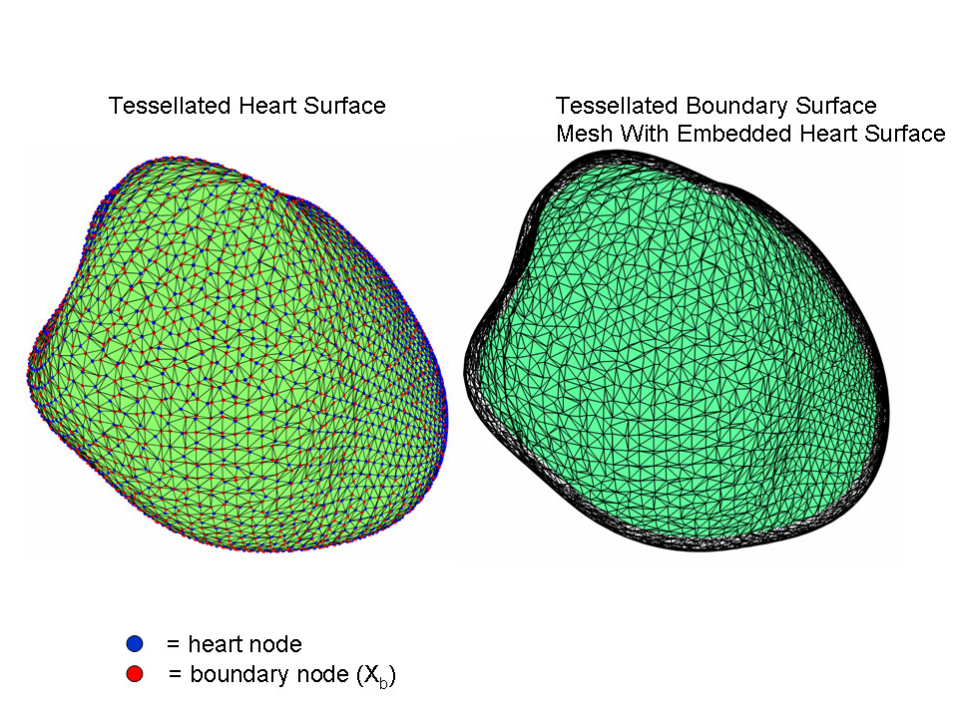
Tessellated surfaces. The heart surface is in the left panel and the boundary surface is in the right panel. The tessellated patterns for the surfaces are identical. In the right hand panel, the boundary surface is represented by a transparent mesh that shows the embedded heart surface (aqua).

The left panel in Figure 6 shows the surface of the example problem heart with both the original heart surface points (blue) and projected grid boundary points X_b _(red) triangulated in the manner described above. The boundary surface (right hand panel in Figure) has shape very similar to that of the heart surface and the triangulation is identical to that shown for the heart surface.

Because each triangle on the heart surface has a corresponding triangle on the boundary surface, connecting the corresponding triangle nodes by radial line segments defines a prismatic element. All of the prismatic elements define the scaffolding mesh.

### Heart to torso transfer matrix

The finite element stiffness matrix (**A**^k^) for the prismatic element (scaffolding) mesh describes the electrical coupling between the heart surface and the boundary surface. However, this matrix contains extraneous elements, i.e. the projected heart nodes on the boundary surface (X_hp_), and the projected boundary nodes on the heart surface (X_bp_). These extraneous nodes must be removed, resulting in a transfer matrix between the heart nodes and the boundary nodes.

The concept of extraneous nodes will be further described with reference to Figure 5. The desired final matrix should reflect only electrical coupling between the original heart mesh nodes (X_h_) shown as filled black circles and the boundary nodes (X_b_) shown as filled red circles. The positions of the boundary nodes (X_b_) are approximated by the dotted red circles (X_b'_), so in actuality the final desired stiffness matrix will reflect the coupling between the filled black circles and the dotted red circles. However, the scaffolding matrix also contains electrical coupling between the desired nodes (filled black and dotted red circles) and extraneous nodes (circles with black and red diagonal lines), which are the projected heart nodes on the boundary surface (X_hp_), and the projected boundary nodes on the heart surface (X_bp_). These extraneous nodes must not exist in the desired final system matrix.

First, to assemble the finite element stiffness matrix (**A**^k^) for the prismatic element mesh, the local stiffness matrix for each prism element is constructed. This may be done in a number of ways. For a Galerkin type FEM, a closed form local matrix based on the prismatic geometry may be used. Alternatively, each prism may be decomposed into tetrahedrons, and the stiffness matrix, based on linear basis functions, may be computed for each tetrahedron.

To remove the extraneous nodes in **A**^k^, it was partitioned as follows:

Ak=[AhhkAhekAhb′kAehkAeekAeb′kAb′hkAb′ekAb′b′k]     (3)
 MathType@MTEF@5@5@+=feaafiart1ev1aaatCvAUfKttLearuWrP9MDH5MBPbIqV92AaeXatLxBI9gBaebbnrfifHhDYfgasaacH8akY=wiFfYdH8Gipec8Eeeu0xXdbba9frFj0=OqFfea0dXdd9vqai=hGuQ8kuc9pgc9s8qqaq=dirpe0xb9q8qiLsFr0=vr0=vr0dc8meaabaqaciaacaGaaeqabaqabeGadaaakeaaieqacqWFbbqqdaahaaWcbeqaaiabdUgaRbaakiabg2da9maadmaabaqbaeqabmWaaaqaaGqadiab+feabnaaDaaaleaacqWGObaAcqWGObaAaeaacqWGRbWAaaaakeaacqWFbbqqdaqhaaWcbaGaemiAaGMaemyzaugabaGaem4AaSgaaaGcbaGae8xqae0aa0baaSqaaiabdIgaOjqbdkgaIzaafaaabaGaem4AaSgaaaGcbaGae8xqae0aa0baaSqaaiabdwgaLjabdIgaObqaaiabdUgaRbaaaOqaaiab=feabnaaDaaaleaacqWGLbqzcqWGLbqzaeaacqWGRbWAaaaakeaacqWFbbqqdaqhaaWcbaGaemyzauMafmOyaiMbauaaaeaacqWGRbWAaaaakeaacqWFbbqqdaqhaaWcbaGafmOyaiMbauaacqWGObaAaeaacqWGRbWAaaaakeaacqWFbbqqdaqhaaWcbaGafmOyaiMbauaacqWGLbqzaeaacqWGRbWAaaaakeaacqWFbbqqdaqhaaWcbaGafmOyaiMbauaacuWGIbGygaqbaaqaaiabdUgaRbaaaaaakiaawUfacaGLDbaacaWLjaGaaCzcamaabmaabaGaeG4mamdacaGLOaGaayzkaaaaaa@65D1@

where the subscript h denotes the heart surface nodes X_h_, the subscript e denotes the extraneous nodes (X_hp _∪ X_bp_) to be removed, and the subscript b' denotes the projected boundary nodes X_b'_. The desired coupling matrix (**A**^c^) between X_h _and X_b' _is found by solving for the extraneous nodes in the set of equations **A**^k ^= 0:

Ac=[AhhcAhb′cAb′hcAb′b′c]     (4)
 MathType@MTEF@5@5@+=feaafiart1ev1aaatCvAUfKttLearuWrP9MDH5MBPbIqV92AaeXatLxBI9gBaebbnrfifHhDYfgasaacH8akY=wiFfYdH8Gipec8Eeeu0xXdbba9frFj0=OqFfea0dXdd9vqai=hGuQ8kuc9pgc9s8qqaq=dirpe0xb9q8qiLsFr0=vr0=vr0dc8meaabaqaciaacaGaaeqabaqabeGadaaakeaaieqacqWFbbqqdaahaaWcbeqaaiabdogaJbaakiabg2da9maadmaabaqbaeqabiGaaaqaaiab=feabnaaDaaaleaacqWGObaAcqWGObaAaeaacqWGJbWyaaaakeaacqWFbbqqdaqhaaWcbaGaemiAaGMafmOyaiMbauaaaeaacqWGJbWyaaaakeaacqWFbbqqdaqhaaWcbaGafmOyaiMbauaacqWGObaAaeaacqWGJbWyaaaakeaacqWFbbqqdaqhaaWcbaGafmOyaiMbauaacuWGIbGygaqbaaqaaiabdogaJbaaaaaakiaawUfacaGLDbaacaWLjaGaaCzcamaabmaabaGaeGinaqdacaGLOaGaayzkaaaaaa@4B0A@

where

Ahhc=Ahhk−Ahek(Aeek)−1AehkAhbc=Ahb′k−Ahek(Aeek)−1Aeb′kAb′hc=Ab′hk−Ab′ek(Aeek)−1AehkAb′b′c=Ab′b′k−Ab′ek(Aeek)−1Aeb′k
 MathType@MTEF@5@5@+=feaafiart1ev1aaatCvAUfKttLearuWrP9MDH5MBPbIqV92AaeXatLxBI9gBaebbnrfifHhDYfgasaacH8akY=wiFfYdH8Gipec8Eeeu0xXdbba9frFj0=OqFfea0dXdd9vqai=hGuQ8kuc9pgc9s8qqaq=dirpe0xb9q8qiLsFr0=vr0=vr0dc8meaabaqaciaacaGaaeqabaqabeGadaaakeaafaqaaeabbaaaaeaaieqacqWFbbqqdaqhaaWcbaGaemiAaGMaemiAaGgabaGaem4yamgaaOGaeyypa0Jae8xqae0aa0baaSqaaiabdIgaOjabdIgaObqaaiabdUgaRbaakiabgkHiTiab=feabnaaDaaaleaacqWGObaAcqWGLbqzaeaacqWGRbWAaaGccqGGOaakcqWFbbqqdaqhaaWcbaGaemyzauMaemyzaugabaGaem4AaSgaaOGaeiykaKYaaWbaaSqabeaacqGHsislcqaIXaqmaaGccqWFbbqqdaqhaaWcbaGaemyzauMaemiAaGgabaGaem4AaSgaaaGcbaGae8xqae0aa0baaSqaaiabdIgaOjabdkgaIbqaaiabdogaJbaakiabg2da9iab=feabnaaDaaaleaacqWGObaAcuWGIbGygaqbaaqaaiabdUgaRbaakiabgkHiTiab=feabnaaDaaaleaacqWGObaAcqWGLbqzaeaacqWGRbWAaaGccqGGOaakcqWFbbqqdaqhaaWcbaGaemyzauMaemyzaugabaGaem4AaSgaaOGaeiykaKYaaWbaaSqabeaacqGHsislcqaIXaqmaaGccqWFbbqqdaqhaaWcbaGaemyzauMafmOyaiMbauaaaeaacqWGRbWAaaaakeaacqWFbbqqdaqhaaWcbaGafmOyaiMbauaacqWGObaAaeaacqWGJbWyaaGccqGH9aqpcqWFbbqqdaqhaaWcbaGafmOyaiMbauaacqWGObaAaeaacqWGRbWAaaGccqGHsislcqWFbbqqdaqhaaWcbaGafmOyaiMbauaacqWGLbqzaeaacqWGRbWAaaGccqGGOaakcqWFbbqqdaqhaaWcbaGaemyzauMaemyzaugabaGaem4AaSgaaOGaeiykaKYaaWbaaSqabeaacqGHsislcqaIXaqmaaGccqWFbbqqdaqhaaWcbaGaemyzauMaemiAaGgabaGaem4AaSgaaaGcbaGae8xqae0aa0baaSqaaiqbdkgaIzaafaGafmOyaiMbauaaaeaacqWGJbWyaaGccqGH9aqpcqWFbbqqdaqhaaWcbaGafmOyaiMbauaacuWGIbGygaqbaaqaaiabdUgaRbaakiabgkHiTiab=feabnaaDaaaleaacuWGIbGygaqbaiabdwgaLbqaaiabdUgaRbaakiabcIcaOiab=feabnaaDaaaleaacqWGLbqzcqWGLbqzaeaacqWGRbWAaaGccqGGPaqkdaahaaWcbeqaaiabgkHiTiabigdaXaaakiab=feabnaaDaaaleaacqWGLbqzcuWGIbGygaqbaaqaaiabdUgaRbaaaaaaaa@AD20@

The set of equations **A**^k ^= 0 is somewhat of an oversimplification of the process of eliminating extraneous nodes. More precisely, the right hand side corresponding to each extraneous node is set to 0. To take a simple example, assume it is desired to eliminate a variable x_2. _If the row in the matrix **A**^k ^corresponding to x_2 _is equal to -3x_1 _+ 4x_2 _- 2x_3_, then setting this row equal to 0 and solving for x_2 _yields x_2 _= 1/4(3x_1 _+ 2x_3_). 1/4(3x_1 _+ 2x_3_) is then substituted for x_2 _wherever it appears in any other equation, effectively eliminating x_2 _from the system of equations.

### Total system matrix

The total system matrix comprises a combination of the preexisting FEM heart matrix (**A**^o^), the FEM heart to torso coupling matrix **A**^c^, and the FEM torso matrix **A**^t^.

The FEM torso matrix **A**^t ^is based on a voxel based mesh of the torso. If the voxel torso mesh did not exist *a priori*, it may be generated by extending the preliminary mesh until it approaches the outer torso, as indicated by the green filled circles shown in Figure 3. The outer torso nodes may be found in a number of ways. In the approach adopted, the original torso surface nodes were multiply interpolated with two dimensional Legendre polynomials, resulting in a set of surface indicator functions analogous to the spherical harmonic surface indicator function described with reference to generation of the boundary surface.

The FEM stiffness matrix **A**^t ^for the voxel matrix was computed once again with a Galerkin type scheme with linear basis functions. For voxel meshes, this type of FEM matrix may be generated very quickly, on the order of a few seconds assuming reasonable computer processing speed and memory.

The FEM matrix (**A**^s^) for the entire system is:

As=[AiioAihoAhioAhhc+AhhoAhb′cAb′hcAb′b′c+Ab′b′tAb′gtAgb′tAggt]     (5)
 MathType@MTEF@5@5@+=feaafiart1ev1aaatCvAUfKttLearuWrP9MDH5MBPbIqV92AaeXatLxBI9gBaebbnrfifHhDYfgasaacH8akY=wiFfYdH8Gipec8Eeeu0xXdbba9frFj0=OqFfea0dXdd9vqai=hGuQ8kuc9pgc9s8qqaq=dirpe0xb9q8qiLsFr0=vr0=vr0dc8meaabaqaciaacaGaaeqabaqabeGadaaakeaaieqacqWFbbqqdaahaaWcbeqaaiabdohaZbaakiabg2da9maadmaabaqbaeqabqabaaaaaeaacqWFbbqqdaqhaaWcbaGaemyAaKMaemyAaKgabaGaem4Ba8gaaaGcbaGae8xqae0aa0baaSqaaiabdMgaPjabdIgaObqaaiabd+gaVbaaaOqaaaqaaaqaaiab=feabnaaDaaaleaacqWGObaAcqWGPbqAaeaacqWGVbWBaaaakeaacqWFbbqqdaqhaaWcbaGaemiAaGMaemiAaGgabaGaem4yamgaaOGaey4kaSIae8xqae0aa0baaSqaaiabdIgaOjabdIgaObqaaiabd+gaVbaaaOqaaiab=feabnaaDaaaleaacqWGObaAcuWGIbGygaqbaaqaaiabdogaJbaaaOqaaaqaaaqaaiab=feabnaaDaaaleaacuWGIbGygaqbaiabdIgaObqaaiabdogaJbaaaOqaaiab=feabnaaDaaaleaacuWGIbGygaqbaiqbdkgaIzaafaaabaGaem4yamgaaOGaey4kaSIae8xqae0aa0baaSqaaiqbdkgaIzaafaGafmOyaiMbauaaaeaacqWG0baDaaaakeaacqWFbbqqdaqhaaWcbaGafmOyaiMbauaacqWGNbWzaeaacqWG0baDaaaakeaaaeaaaeaacqWFbbqqdaqhaaWcbaGaem4zaCMafmOyaiMbauaaaeaacqWG0baDaaaakeaacqWFbbqqdaqhaaWcbaGaem4zaCMaem4zaCgabaGaemiDaqhaaaaaaOGaay5waiaaw2faaiaaxMaacaWLjaWaaeWaaeaacqaI1aqnaiaawIcacaGLPaaaaaa@77E0@

where the subscript g denotes the set of torso mesh nodes excluding the boundary nodes, and the subscript i denotes the set of original heart nodes excluding the outer surface heart nodes X_h_. In this equation, the preexisting FEM heart matrix **A**^o ^has been partitioned into components: (i) that connect interior heart nodes to other interior heart nodes (Aiio
 MathType@MTEF@5@5@+=feaafiart1ev1aaatCvAUfKttLearuWrP9MDH5MBPbIqV92AaeXatLxBI9gBaebbnrfifHhDYfgasaacH8akY=wiFfYdH8Gipec8Eeeu0xXdbba9frFj0=OqFfea0dXdd9vqai=hGuQ8kuc9pgc9s8qqaq=dirpe0xb9q8qiLsFr0=vr0=vr0dc8meaabaqaciaacaGaaeqabaqabeGadaaakeaaieqacqWFbbqqdaqhaaWcbaGaemyAaKMaemyAaKgabaGaem4Ba8gaaaaa@3207@); (ii) that connect interior heart nodes to nodes on the heart surface (Ahio
 MathType@MTEF@5@5@+=feaafiart1ev1aaatCvAUfKttLearuWrP9MDH5MBPbIqV92AaeXatLxBI9gBaebbnrfifHhDYfgasaacH8akY=wiFfYdH8Gipec8Eeeu0xXdbba9frFj0=OqFfea0dXdd9vqai=hGuQ8kuc9pgc9s8qqaq=dirpe0xb9q8qiLsFr0=vr0=vr0dc8meaabaqaciaacaGaaeqabaqabeGadaaakeaaieqacqWFbbqqdaqhaaWcbaGaemiAaGMaemyAaKgabaGaem4Ba8gaaaaa@3205@ and Aiho
 MathType@MTEF@5@5@+=feaafiart1ev1aaatCvAUfKttLearuWrP9MDH5MBPbIqV92AaeXatLxBI9gBaebbnrfifHhDYfgasaacH8akY=wiFfYdH8Gipec8Eeeu0xXdbba9frFj0=OqFfea0dXdd9vqai=hGuQ8kuc9pgc9s8qqaq=dirpe0xb9q8qiLsFr0=vr0=vr0dc8meaabaqaciaacaGaaeqabaqabeGadaaakeaaieqacqWFbbqqdaqhaaWcbaGaemyAaKMaemiAaGgabaGaem4Ba8gaaaaa@3205@); and (iii) that connect outer heart nodes to other outer heart nodes (Ahho
 MathType@MTEF@5@5@+=feaafiart1ev1aaatCvAUfKttLearuWrP9MDH5MBPbIqV92AaeXatLxBI9gBaebbnrfifHhDYfgasaacH8akY=wiFfYdH8Gipec8Eeeu0xXdbba9frFj0=OqFfea0dXdd9vqai=hGuQ8kuc9pgc9s8qqaq=dirpe0xb9q8qiLsFr0=vr0=vr0dc8meaabaqaciaacaGaaeqabaqabeGadaaakeaaieqacqWFbbqqdaqhaaWcbaGaemiAaGMaemiAaGgabaGaem4Ba8gaaaaa@3203@). Similarly, **A**^c ^and **A**^t ^have been partitioned into portions: (i) that connect "internal nodes" (i.e. nodes that are not a part of any other matrix) to other "internal nodes"; (ii) that connect "internal nodes" to "overlapping nodes" (i.e. nodes that appear in two matrices); and (iii) that that connect "overlapping nodes" to "overlapping nodes."

For example, Ahho
 MathType@MTEF@5@5@+=feaafiart1ev1aaatCvAUfKttLearuWrP9MDH5MBPbIqV92AaeXatLxBI9gBaebbnrfifHhDYfgasaacH8akY=wiFfYdH8Gipec8Eeeu0xXdbba9frFj0=OqFfea0dXdd9vqai=hGuQ8kuc9pgc9s8qqaq=dirpe0xb9q8qiLsFr0=vr0=vr0dc8meaabaqaciaacaGaaeqabaqabeGadaaakeaaieqacqWFbbqqdaqhaaWcbaGaemiAaGMaemiAaGgabaGaem4Ba8gaaaaa@3203@ represents the coupling between the outer heart surface nodes, which are overlapping nodes, to themselves in the matrix **A**^*o*^. Similarly, Ahhc
 MathType@MTEF@5@5@+=feaafiart1ev1aaatCvAUfKttLearuWrP9MDH5MBPbIqV92AaeXatLxBI9gBaebbnrfifHhDYfgasaacH8akY=wiFfYdH8Gipec8Eeeu0xXdbba9frFj0=OqFfea0dXdd9vqai=hGuQ8kuc9pgc9s8qqaq=dirpe0xb9q8qiLsFr0=vr0=vr0dc8meaabaqaciaacaGaaeqabaqabeGadaaakeaaieqacqWFbbqqdaqhaaWcbaGaemiAaGMaemiAaGgabaGaem4yamgaaaaa@31EB@ represents the coupling between the outer heart surface nodes to themselves in the matrix **A**^*c*^. The matrix entry in **A**^s ^for the coupling of the outer heart nodes to themselves is thus the combined entry Ahho
 MathType@MTEF@5@5@+=feaafiart1ev1aaatCvAUfKttLearuWrP9MDH5MBPbIqV92AaeXatLxBI9gBaebbnrfifHhDYfgasaacH8akY=wiFfYdH8Gipec8Eeeu0xXdbba9frFj0=OqFfea0dXdd9vqai=hGuQ8kuc9pgc9s8qqaq=dirpe0xb9q8qiLsFr0=vr0=vr0dc8meaabaqaciaacaGaaeqabaqabeGadaaakeaaieqacqWFbbqqdaqhaaWcbaGaemiAaGMaemiAaGgabaGaem4Ba8gaaaaa@3203@ + Ahhc
 MathType@MTEF@5@5@+=feaafiart1ev1aaatCvAUfKttLearuWrP9MDH5MBPbIqV92AaeXatLxBI9gBaebbnrfifHhDYfgasaacH8akY=wiFfYdH8Gipec8Eeeu0xXdbba9frFj0=OqFfea0dXdd9vqai=hGuQ8kuc9pgc9s8qqaq=dirpe0xb9q8qiLsFr0=vr0=vr0dc8meaabaqaciaacaGaaeqabaqabeGadaaakeaaieqacqWFbbqqdaqhaaWcbaGaemiAaGMaemiAaGgabaGaem4yamgaaaaa@31EB@.

The discretized version of equation (1) is **A**^s^**V**_e _= **A**^o, rhs^**V**_t_, where **V**_e _and **V**_t _are the vectors corresponding to the continuous variables in equation (1), and **A**^o, rhs ^is the original, pre-computed stiffness matrix corresponding to the right hand side of equation (1). **A**^o, rhs ^and **V**_t _must be appropriately augmented with 0's to match the size of **A**^s^**V**_e_. The boundary conditions at the heart/torso boundary, that V_e _is continuous and the normal component of the current is continuous[3], are necessarily enforced by the finite element formulation used to create **A**^s^.

### Test

The above described scheme was tested as follows. A spherical shell, analogous to the outer heart surface, was coupled to a surrounding spherical "torso". The potentials on the inner spherical shell were set equal to those which would have occurred had there been an ideal current dipole, normalized to unit strength, located at the sphere's origin and oriented along the z axis. Given this potential distribution, the potentials throughout the spherical "torso" were computed and compared with the analytic solution[8]:

V(r,ϕ)=cos⁡(ϕ)∗(1r2+2Rr3)     (6)
 MathType@MTEF@5@5@+=feaafiart1ev1aaatCvAUfKttLearuWrP9MDH5MBPbIqV92AaeXatLxBI9gBaebbnrfifHhDYfgasaacH8akY=wiFfYdH8Gipec8Eeeu0xXdbba9frFj0=OqFfea0dXdd9vqai=hGuQ8kuc9pgc9s8qqaq=dirpe0xb9q8qiLsFr0=vr0=vr0dc8meaabaqaciaacaGaaeqabaqabeGadaaakeaacqWGwbGvcqGGOaakcqWGYbGCcqGGSaaliiGacqWFvpGAcqGGPaqkcqGH9aqpcyGGJbWycqGGVbWBcqGGZbWCcqGGOaakcqWFvpGAieaacqGFPaqkcqGHxiIkdaqadaqaamaalaaabaGaeGymaedabaGaemOCai3aaWbaaSqabeaacqaIYaGmaaaaaOGaey4kaSYaaSaaaeaacqaIYaGmcqWGsbGuaeaacqWGYbGCdaahaaWcbeqaaiabiodaZaaaaaaakiaawIcacaGLPaaacaWLjaGaaCzcamaabmaabaGaeGOnaydacaGLOaGaayzkaaaaaa@4BD9@

where Φ is the polar angle (as before), R is the radius of the outer spherical torso surface, and r is the radius to an observation point. For the test problem, R was set at 50. The radius of the spherical shell/heart surface was set at 40, roughly the same size as the heart shown in Figure 1. To create the potential distribution equivalent to a dipole oriented along the z axis, the potentials on this sphere/heart surface were thus set at:

V(40,ϕ)=cos⁡(ϕ)∗(1402+2∗50403).     (7)
 MathType@MTEF@5@5@+=feaafiart1ev1aaatCvAUfKttLearuWrP9MDH5MBPbIqV92AaeXatLxBI9gBaebbnrfifHhDYfgasaacH8akY=wiFfYdH8Gipec8Eeeu0xXdbba9frFj0=OqFfea0dXdd9vqai=hGuQ8kuc9pgc9s8qqaq=dirpe0xb9q8qiLsFr0=vr0=vr0dc8meaabaqaciaacaGaaeqabaqabeGadaaakeaacqWGwbGvcqGGOaakcqaI0aancqaIWaamcqGGSaaliiGacqWFvpGAcqGGPaqkcqGH9aqpcyGGJbWycqGGVbWBcqGGZbWCcqGGOaakcqWFvpGAcqGGPaqkcqGHxiIkdaqadaqaamaalaaabaGaeGymaedabaGaeGinaqJaeGimaaZaaWbaaSqabeaacqaIYaGmaaaaaOGaey4kaSYaaSaaaeaacqaIYaGmcqGHxiIkcqaI1aqncqaIWaamaeaacqaI0aancqaIWaamdaahaaWcbeqaaiabiodaZaaaaaaakiaawIcacaGLPaaacqGGUaGlcaWLjaGaaCzcamaabmaabaGaeG4naCdacaGLOaGaayzkaaaaaa@4FC7@

A voxel mesh, akin to the torso mesh shown in Figure 3, was generated to fill in the space between the inner and outer spherical surfaces, and the corresponding FEM matrix **A**^t ^was computed. Next, a coupling matrix A^c ^to link the torso to the nodes on the spherical shell/heart surface was created. The system of equations **A**^s ^*V_c _= 0 was constructed, where **A**^s ^is the total system FEM matrix and **V**_c _is the solution. The spherical shell/heart surface nodes were set equal to their corresponding potential V(40, Φ), resulting in an augmented system **A**'*V' = **b**, where **V**(i)' = 1 where i represents the spherical shell/heart surface nodes. This system was solved using the biconjugate gradient stable method, after preconditioning by scaling each row in the matrix so that its diagonal element was equal to 1.

Relative error was defined as:

RE=∑(Vc−Va)2∑Va2     (8)
 MathType@MTEF@5@5@+=feaafiart1ev1aaatCvAUfKttLearuWrP9MDH5MBPbIqV92AaeXatLxBI9gBaebbnrfifHhDYfgasaacH8akY=wiFfYdH8Gipec8Eeeu0xXdbba9frFj0=OqFfea0dXdd9vqai=hGuQ8kuc9pgc9s8qqaq=dirpe0xb9q8qiLsFr0=vr0=vr0dc8meaabaqaciaacaGaaeqabaqabeGadaaakeaacqWGsbGucqWGfbqrcqGH9aqpdaGcaaqaamaalaaabaWaaabqaeaacqGGOaakcqWGwbGvdaWgaaWcbaGaem4yamgabeaakiabgkHiTiabdAfawnaaBaaaleaacqWGHbqyaeqaaOGaeiykaKYaaWbaaSqabeaacqaIYaGmaaaabeqab0GaeyyeIuoaaOqaamaaqaeabaGaemOvay1aa0baaSqaaiabdggaHbqaaiabikdaYaaaaeqabeqdcqGHris5aaaaaSqabaGccaWLjaGaaCzcamaabmaabaGaeGioaGdacaGLOaGaayzkaaaaaa@44DB@

where V_c _is the computed solution (excluding the spherical shell/heart nodes) and V_a _is the analytical solution. Both the number of nodes on the spherical shell/heart surface and the number of nodes in the "torso" were varied.

### Heart beat

The heart in Figure 1, which is based on the Auckland canine model[2], was meshed[1] and oriented within a human shaped torso, as shown. A cellular automata like scheme was used to generate an activation and repolarization sequence throughout the heart, resulting in a corresponding sequence of transmembrane potentials *V*_*t*_. Maximum *V*_*t *_was 100 mV, with the resting potential set at 0 mV. Initial left ventricular and right ventricular activation sites were chosen roughly in accord with Selvester et al.[9] but were adjusted to generate better matches of epicardial potentials with recordings made by Spach et al.[10] in intact chimpanzees.

A propagation sequence was generated by a simple eikonal like scheme, according to which propagation velocity (v) was assumed to be proportional to the square root of bulk cardiac tissue conductivity[11]. The eikonal like scheme was implemented by calculating a propagation time between connected nodes in the heart mesh, selecting initial activation sites, and then stepping through time to activate the remaining heart nodes. The activation time of a heart node was set equal to the activation time of its nearest neighbor plus the approximate propagation time from the heart node to this nearest neighbor. The propagation time between neighboring nodes was set equal to v/d, where v is velocity (proportional to the square root of the conductance between nodes) and d is the distance between nodes.

Repolarization was initiated from the right ventricular breakthrough area[10] and allowed to spread like a wave across the epicardium in the manner in which activation was simulated. Repolarization was then allowed to proceed inward transmurally toward the epicardium based simply on relative transmural depth. In other words, repolarization proceeded from the epicardium inward. Although this oversimplified repolarization sequence is not necessarily strictly physiologically correct[12], it was chosen because of convenience and because it produced reasonable intra-epicardial gradients and epicardial/endocardial transmembrane gradients that resulted in realistic T waves.

Within the heart muscle: (i) intracellular conductivity along and transverse to fiber direction was set equal to 1 and 1/6, respectively; (ii). extracellular conductivity along and transverse to fiber direction was set equal to 1 and 1/3, respectively. Ventricular blood and torso conductivity were set at 5 and 2.5, respectively.

The heart mesh had approximately 270000 nodes and the torso mesh had approximately 120000 nodes. The system of equations was solved with Matlab's preconditioned conjugate gradient routine. The preconditioner was an incomplete Cholesky (0 fill in) factorization of the system matrix.

## Results

**Figure 7:**
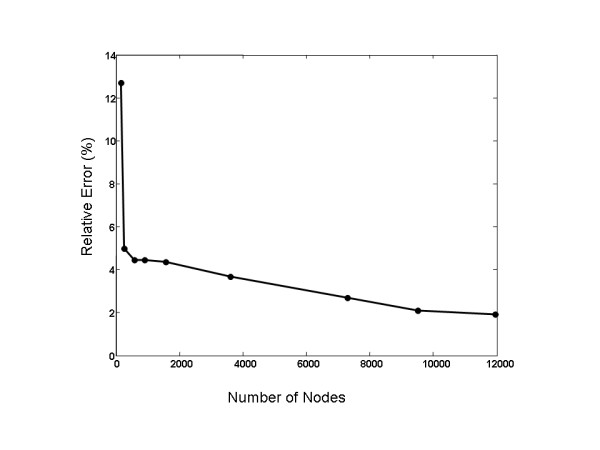
Results of the sphere test. Convergence is not monotonic and is relatively slow after approximately a 4% error has been reached.

For the sphere test, Figure 7 shows relative error as a function of the number of total nodes, i.e. heart surface nodes (inner spherical shell) and torso nodes (between inner and outer spherical shells). The error drops rapidly to somewhat over 4% but convergence is markedly slower thereafter. It should be noted that different error curves were obtained depending on the relative balance between heart surface nodes and torso nodes; the curve shown in Figure 7 is representative of the general pattern of fast convergence with relatively few nodes and slow convergence after that. If the number of heart surface nodes (inner spherical shell) was held constant and the number of torso nodes increased, convergence was not necessarily monotonic.

Figure 1 shows the resulting simulated electrocardiograms at various locations roughly corresponding to the 6 precordial leads in the standard 12 lead electrocardiogram. The electrograms have a reasonably realistic shape. The peak to peak amplitude of approximately 4–5 mV is in line with the approximately 4.5 mV voltage difference across the torso in a body surface mapping study of chimpanzees[10]. The simulated maximum voltage difference across the epicardium (which occurred approximately 40 ms into the QRS complex) was approximately 13 mV, which is similar to the maximum epicardial potential difference reported by Spach et al. 28 ms into the QRS onset of the chimpanzee QRS complex.

**Figure 8:**
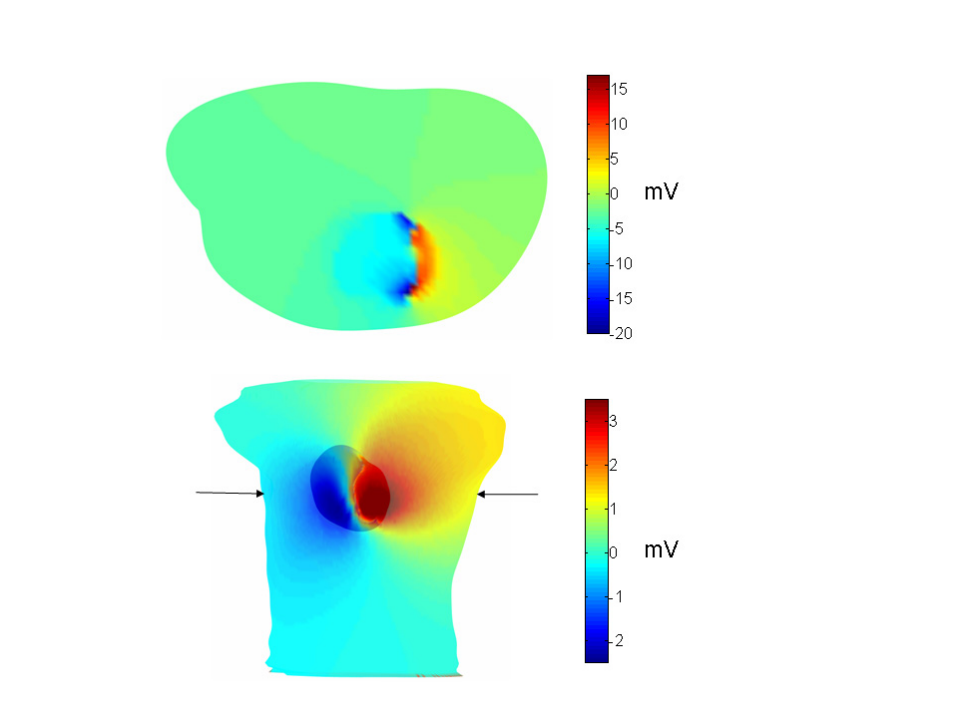
Computed potentials. Potentials on the heart and torso are shown in the lower panel and potentials through the cross section indicated by arrows are shown in the upper panel. In the lower panel, the heart potentials, which are greater than the torso potentials, saturate the color scheme.

The top panel in Figure 8 is a cross sectional view through the torso, along the plane indicated in the lower panel, which also shows the potentials on the torso surface and the outer heart surface. (In the lower panel, the colorbar is scaled to the torso potentials; the larger magnitude maximum and minimum heart potentials are saturated in this color scale and are simply red and blue, respectively.) The positive and negative tissue on the outer heart surface is faithfully projected onto the torso surface (bottom panel), as desired. The transverse slice shows a potential jump from the depolarized outer heart surface to the adjacent torso volume, as theory predicts[13]. The potential distribution across the torso slice appears to be reasonable.

## Discussion

This work describes a convenient scheme for coupling a pre-existing, possibly curvilinear, finite element mesh to a voxel mesh. The scheme is easy to code and generates a complete system matrix (i.e. the coupling and torso matrices along with final matrix assembly) fairly quickly, on the order of 10's of seconds for the heart/torso coupling example problem on a 2.80 GHz Xeon processor running Microsoft Windows XP with 3 GB of DRAM. The relatively good speed is helpful in cases, such as in the example problem, where some experimentation is required to fix the orientation of the preexisting and voxel meshes.

In this problem, the only user supervision involved orienting the heart within the torso. The rest of the process, namely the generation of coupling and torso matrices and assembly of the final matrix, was automatic.

The disadvantages of the scheme are that it appears to converge relatively slowly after a given degree of accuracy has been reached. It also may converge non-monotonically if only the number of nodes in the voxel mesh is increased. Given the weight of advantages and disadvantages, the scheme may be most useful for prototyping, where high accuracy is not required at the outset and experimentation is required to align the preexisting and voxel meshes.

One possible reason for the slow convergence is that the boundary surface S_b _can not perfectly fit the boundary nodes because this surface can not follow the sharp corners between some of the adjacent boundary nodes. Indeed, for this reason, the error of the fit between S_b _and the boundary nodes decreases slowly as the number of torso nodes is increased, which mirrors the overall slow convergence of the scheme.

There are a few possibilities for improving the scheme. First, instead of trying to fit the boundary surface to all of the boundary nodes (Figure 3), the corner boundary nodes (at the vertices of the rectangle defined blue red circles in Figure 3) could be excluded from the surface fit, yielding a better surface fit to the non-corner boundary nodes. These corner nodes could be coupled to the boundary surface through tetrahedral elements.

**Figure 9:**
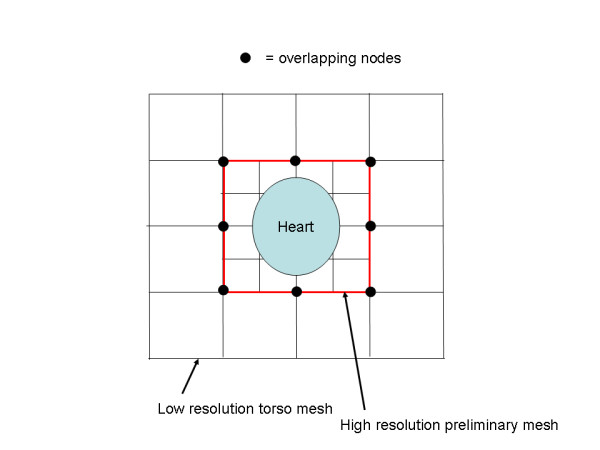
Alternative meshing scheme. A high resolution scaffolding mesh is coupled to a low resolution torso mesh.

Another possible improvement would be to generate the coupling matrix with a very high spatial resolution mesh, and then couple this to a lower spatial resolution voxel mesh by removing extraneous nodes from the higher resolution mesh in the same manner that extraneous nodes were removed from the scaffolding matrix in the scheme described above. The coupling between higher and lower resolution meshes would be easy because both are voxel based meshes, so that it is easy to generate a low resolution mesh whose nodes are all within the set of the high resolution mesh nodes. For example, the high resolution mesh could be generated by creating nodes at the midpoints of the low resolution mesh, as shown in Figure 9. This mesh is non-conforming, due to the presence of nodes in the smaller elements that are not nodes of the neighboring larger elements. As an alternative to removing these nodes through elimination, which may cause numerical problems, this type of mesh may be treated in the manner described by Schimpf et al.[14]

Yet another alternative would be to couple the outer heart surface and the boundary surface with the boundary element method, which would avoid the requirement of eliminating extraneous nodes.

The present scheme may be applied to problems in bioengineering other than the heart/torso coupling problem. Further, the present scheme could be used in areas outside of bioengineering. However, extensions of the scheme to problems in which the preexisting mesh (e.g. the heart mesh) is not reasonably smooth may prove difficult because prismatic elements in the scaffolding matrix could be very irregularly shaped. Similarly, irregular node spacing on the preexisting mesh might also cause problems. If there are too few nodes on the outer surface of the preexisting mesh, then the scaffolding mesh might not have sufficient resolution for an accurate solution. If the physics of a problem require a very accurate representation of the geometry in the area of the boundary between the scaffolding mesh and voxel mesh, the present scheme will likely not prove suitable.

In summary, a scheme has been advanced that may be useful in situations where rapid computational speed and ease of use is a higher priority than great accuracy. Further, it may be possible to improve the convergence of the present scheme.

## Glossary

**A**^c ^– the desired coupling matrix between X_h _and X_b'_

**A**^k ^– the FEM scaffolding mesh matrix

**A**^o ^– the preexisting FEM heart matrix

**A**^s ^– the total system matrix, assembled from **A**^o^, **A**^c^, and **A**^t^

**A**^t ^– the FEM torso matrix

Boundary nodes – the nodes within the preliminary voxel mesh that are closest to, but outside of, the outer heart surface

Boundary surface – a surface that is fitted to the boundary nodes

Outer heart surface – the outer surface of the preexisting heart mesh

Preliminary voxel mesh – a voxel mesh that is slightly larger than the heart mesh, which is embedded within the preliminary voxel mesh

Scaffolding mesh – a mesh that is constructed that fills in the volume between the outer heart surface and the boundary surface

S_h _– outer heart surface

S_b _– boundary surface

X_p _– nodes in the preliminary voxel mesh

X_b _– boundary nodes

R_h _– radius of spherical harmonic surface that approximates S_h_

X'_p _– X_p _points translated to the origin of the heart coordinate system

X_h _– the subset of X_p _that lies within the heart

X_b' _– nodes derived by projecting boundary nodes on to S_b_

X_hp _– the heart nodes as projected on the boundary surface; part of scaffolding mesh nodes

X_bp _– the boundary nodes as projected on the heart surface; part of scaffolding mesh nodes
